# Conducting Delphi surveys in medical education research

**DOI:** 10.3205/zma001800

**Published:** 2026-01-15

**Authors:** Angelika Homberg

**Affiliations:** 1 Medical Faculty Mannheim, Heidelberg University, Division for Study and Teaching Development, Department of Medical Education Research, Mannheim, Germany

**Keywords:** Delphi technique, education research, program development, research design, knowledge discovery

## Abstract

**Background:**

Delphi surveys are becoming increasingly important in medical education research, particularly in the development of curricula, assessment instruments, and recommendations for action. However, due to the flexibility and low standardisation of the method, researchers are faced with the challenge of making numerous methodological decisions before and during a Delphi survey. To ensure a structured and targeted approach, careful planning is essential prior to conducting a Delphi study.

**Planning Delphi studies:**

This article describes how to plan Delphi surveys in the following five steps: 1. Suitability and feasibility of the method, 2. Research question and persons involved, 3. Planning of the survey process up to the first round, 4. Evaluation strategies and planning the follow-up rounds, 5. Presentation and dissemination of the results. Each step is structured on the basis of central questions. The most important aspects are summarised in a checklist.

**Conclusion:**

This guide provides researchers with a comprehensive overview of the methodological possibilities and limitations of Delphi surveys, highlighting potential pitfalls. It supports strategic planning and helps researchers to make informed decisions. In the long run, the quality of Delphi studies in medical education research can thus be improved, enabling the method's potential to be realised more effectively.

## Zusammenfassung

**Hintergrund:**

Delphi-Befragungen gewinnen in der medizinischen Ausbildungsforschung an Bedeutung, insbesondere für die Entwicklung von Curricula, Assessmentinstrumenten oder Handlungsempfehlungen. Aufgrund der Flexibilität und geringen Standardisierung der Methode stehen Forschende vor der Herausforderung, zahlreiche methodische Entscheidungen vor und im Verlauf einer Delphi-Befragung zu treffen. Um ein strukturiertes und zielgerichtetes Vorgehen sicherzustellen, ist eine sorgfältige Planung vor Beginn einer Delphi-Studie unerlässlich.

**Planung von Delphi-Studien:**

Die Planung von Delphi-Befragungen wird in diesem Beitrag in folgenden fünf Schritten beschrieben: 1. Eignung und Durchführbarkeit der Methode, 2. Forschungsfrage und beteiligte Personen, 3. Planung des Befragungsprozesses bis zur ersten Runde, 4. Auswertstrategien und Planung der Folgerunden, 5. Darstellung und Verbreitung der Ergebnisse. Jeder Schritt wird anhand zentraler Fragen strukturiert. Die jeweils wichtigsten Punkte werden in einer Checkliste zusammengefasst.

**Schlussfolgerung:**

Dieser Leitfaden bietet Forschenden einen umfassenden Überblick über die methodischen Möglichkeiten und Grenzen von Delphi-Befragungen und weist auf mögliche Fallstricke hin. Er unterstützt bei der vorausschauenden Planung und hilft, fundierte Entscheidungen zu treffen. Langfristig kann dadurch die Qualität von Delphi-Studien in der medizinischen Ausbildungsforschung gesteigert und das Potenzial der Methode besser ausgeschöpft werden.

## 1. Introduction

Medical education research is a broad field that includes the development of consensus-based normative guidelines and recommendations [1]. In this context, Delphi surveys are becoming increasingly important, as they facilitate structured communication between experts, ultimately yielding reliable, differentiated results [2], [3]. They are characterised by the following features: Experts are interviewed anonymously using standardised questionnaires, often with open-ended questions to capture arguments, in at least two rounds. The statistical analysis is usually based on descriptive data. From the second round onwards, the experts receive feedback on the previous round´s results and can thus reconsider and, if necessary, revise their assessments. The questionnaire can also be adapted based on the respondents’ feedback [4], [5], [6]. 

Delphi surveys can be used for both qualitative and quantitative analyses. They can be adapted to different contexts and thus used in a variety of ways. They are particularly suitable for dealing with complex issues where knowledge is uncertain or in need of interpretation [6]. As the process unfolds, diverse perspectives can be considered and a consensus on a given task can be achieved [7], [8]. 

Tasks in medical education research include:

- revising or developing curricula (e.g. [9], [10]),
- defining graduate profiles, competencies or learning objectives (e.g. [11], [12], [13]),
- the revision or development of items for assessment tools (e.g. [14], [15], [16]),
- developing outcome-based indicators for assessing learning outcomes (e.g. [17], [18]),
- collecting and evaluating of ideas and influencing factors for further developing innovative areas (e.g. [19], [20]), and
- conducting needs analyses (e.g. [21], [22]).

Delphi surveys are currently conducted using a variety of different concepts, methodological foundations and quality levels [23], [24]. The term *modification* is used inconsistently [7], [25], [26]. Face-to-face meetings are often incorporated into the survey process to increase the likelihood of the results being applied in practice through direct interaction [27], [28]. In this case, the participants are no longer anonymous. 

Due to their complexity, Delphi surveys require the entire research process to be carefully planned. The flexibility of the methodology and the lack of standardisation place greater demands on researchers. In his 2014 standard work, “Delphi-Befragungen”, Häder describes some important aspects in detail, focusing on social science and futurology [29]. The currently existing recommendations for conducting Delphi surveys in the health sciences (e.g. [23], [25], [30], [31]) and in medical education research (e.g. [32], [33], [34]) were published before 2020. Due to technical and epistemological developments, the methodology has become more specialised in recent years. The DFG-funded DeWiss network (Delphi techniques in health and social sciences) has also contributed to this. As well as addressing specific applications, the network considered fundamental questions relating to the Delphi method and conducted an international Delphi study to develop comprehensive reporting guidelines for the health and social sciences [35].

This “how to” guide aims to provide researchers conducting Delphi surveys in medical education with an overview of the possibilities and challenges of this method. This will enable them to identify potential pitfalls early on and produce valid, well-founded results. Researchers are guided through the five-step process using guiding questions. Finally, a checklist summarising all the important points is provided (see attachment 1 ). Experienced researchers can also use this “how to” for targeted and structured planning.

## 2. Planning a Delphi survey

### 2.1. Step 1: Suitability and feasibility of the method

#### 2.1.1. Is the Delphi survey the most appropriate method?

Firstly, the specific problem to be investigated must be clarified. Whether the Delphi method is suitable depends primarily on the framework conditions surrounding the problem, rather than the question itself [6]. Table 1 (Tab. 1) lists aspects that support the use of the Delphi method, based on Linstone and Turoff [36]. 

If none or very few of these aspects apply, consider whether other consensus methods, such as a consensus conference or the nominal group technique, would be more suitable. As another option, a group discussion, expert survey or workshop could be more appropriate [37]. 

#### 2.1.2. What resources are available?

The complexity of the survey significantly impacts the amount of resources required. However, with good planning, Delphi surveys can be carried out independently by relatively small research groups within a few months. Conducting the survey as part of a doctoral thesis is also feasible [38]. 

The time resources required are often underestimated. For each survey round, time slots should be scheduled for follow-up recordings of non-respondents and for periods of high workload (e.g. examination periods) and holidays. Around eight to ten weeks per round is realistic. Between survey rounds, the questionnaires must be evaluated, feedback prepared, and the next round's questionnaire developed and pre-tested quickly, in order to maintain participants' motivation and reduce dropout rates.

Financial resources may be required for incentives, workshops (e.g. room rental and travel costs), producing accompanying materials, and licences for survey tools and programmes for evaluating the data [39]. Simple Delphi surveys can use standard survey tools that guarantee participant anonymity and data security (e.g. [40]). For more complex procedures, specialised tools are worthwhile because they automate processes such as administration, evaluation, and providing feedback on the data. They also provide templates and enable multi-channel surveys. Real-time calculations of group results can be carried out using individual tools as part of a Real-Time Delphi study [41]. 

#### 2.1.3. When are Delphi variants used?

In recent decades, various methodological variants have been established [8], which override individual typical features of a standard Delphi survey. For example, *Real-Time Delphi* [41] have no distinct Delphi rounds. In *Group Delphi*, the anonymity of the experts is lifted [42] while *Deliberative Delphi* involves citizens who have been trained by experts are involved instead of the experts themselves [43]. Using a Real-Time or Group Delphi can significantly reduce the required time. In medical education research, the Delphi method is particularly useful for achieving consensus on complex issues through synchronous discussion. Deliberative Delphi studies involving patients (e.g., [44]) or students (e.g., [11], [45], [46]) can also be useful for addressing specific concerns. The publication by Niederberger and Deckert provides a good overview of the characteristics of the currently established variants [8]. 

### 2.2. Step 2: Research question and persons involved

#### 2.2.1. Who is involved in planning and managing the process? 

To make well-founded and objective decisions associated with a Delphi survey, a steering group should be formed. Typically, steering group members do not participate in the Delphi survey. One exception is the participatory approach, in which steering group members can also form the expert panel (e.g. [44]). 

In general, the following people can be considered for the steering group:

- Researchers conducting the Delphi survey.
- Researchers who have experience with the Delphi method.
- Researchers who are familiar with the background of the problem to be investigated.
- Decision-makers, stakeholders, and others, such as deans of studies (strategic approach).
- Those affected, such as students or patients (participatory approach).

Using a strategic or participatory approach can increase the acceptance of the results or their adaptation to specific framework conditions. 

It can be useful to involve auxiliary staff to help manage the multiple tasks [29]. It is important to clearly communicate the areas of responsibility and task allocation within the steering group and to consistently document the planning and the decisions made throughout the process. 

#### 2.2.2. How is the research question determined?

The first task of the steering group is to review the research objective and formulate a precise research question. This ensures that all members have the same understanding of the research subject and objective, preventing misunderstandings. 

The following should be considered:

- whether technical terms are used and understood equally by all participants;
- whether the results should only be applicable to a specific geographic region, nationally or internationally;
- under what conditions the results are to be applied (e.g. in schools, universities or other educational institutions).
- The overall methodological objective to be achieved should also be considered (e.g. consensus building, idea aggregation or collection of expert opinions).

In medical education research, Delphi surveys are the predominant method for building consensus. The steering group should verify whether other objectives, such as idea aggregation or the collection of expert opinions, are prioritised. Häder provides a good overview of the respective differentiation [29]. 

#### 2.2.3. Which groups of people are surveyed? 

In Delphi studies, respondents are usually referred to as experts: informed individuals or specialists with knowledge in a specific field [25]. As reflective practitioners, they contribute their real-life experiences to the Delphi process [27]. The composition of the expert panel largely depends on the methodological objective of the Delphi survey [29]. 

In consensus Delphi studies, the population diversity of participants who have relevant expertise on the research topic should be represented. All relevant groups should be integrated into the Delphi process in the most balanced way possible [47], [48] (see table 2 (Tab. 2)), with each group represented by at least two people, preferably three. In this way, different points of view within the respective group can be recorded and a dropout of individual persons has less influence on the overall result. In principle, additional experts can be recruited later on. This should be considered, in particular, if dropping out could lead to distorted results (e.g., if a disproportionate number of experts leave a group) or reduce statistical significance due to a smaller sample size (e.g., in small samples). In order to maintain the comparability, consistency and integrity of the study, the new participants should have comparable expertise and be familiarised with the previous results and the course of the study. The researchers must disclose and justify the subsequent recruitment. 

One of the major difficulties is defining what constitutes an expert [49]. The steering group should therefore discuss this issue in relation to the objectives of the Delphi survey, since the composition of the expert panel has a significant influence on the reliability and validity of the study.

#### 2.2.4. How is the sample size determined? 

As complete enumeration is generally not possible with Delphi surveys, a sample must be selected. Two factors are particularly relevant to the success of the survey: the respondents' individual expertise in relation to the task and their personal interest in the topic. Targeted selection can significantly reduce dropout rates [50]. The following criteria can be used to proceed systematically: Number of published articles on the subject area in question or publication index, participation in specialist committees, professional role or activities, active participation in relevant networks and forums. Faculties or universities may also be asked to nominate experts in specific subject areas. Students can be recruited via the student council, for example, and patients via self-help groups. The personal contacts and affiliations of the steering group members with relevant networks can be helpful in identifying motivated individuals.

#### 2.2.5. How many experts should be included?

There are no uniform recommendations on how many experts should be included. According to Turoff [51], a group of ten to forty people is favorable. Trevelyan et al. suggest eight to fifteen participants for homogeneous panels in the field of health sciences [30]. Delphi surveys in medical education research use panels ranging in size from eight participants (e.g. [38]) to over one hundred participants (e.g. [52]).

The time required increases with the number of participants, especially if the survey contains a high percentage of qualitative questions and the steps are not automated.

A small expert panel is sufficient under the following conditions:

- Only a few topics, theses and questions need to be dealt with.
- Only a few different expert opinions, viewpoints and perspectives need to be taken into account.
- There is a small population that can be mapped.
- The Delphi survey can be carried out in a short period of time.
- The motivation of the participants, and thus the expected response rate, is estimated to be rather high.
- The expected willingness of participants to contribute arguments and ideas is considered to be high.
- It is possible to recruit replacement experts after the first round.

### 2.3. Step 3: Planning the survey process up to the first round

#### 2.3.1. How are the items for the first questionnaire developed?

In the field of educational research, Garavalia et al. [53], following Linstone [36], describe the classic Delphi procedure as consisting of three rounds.

- *First round:* Collection of items based on an open-ended question.
- *Second round: *Evaluation of the collected items using rating scales and arguments captured through open-ended questions.
- *Third round: *Re-evaluation of the items based on feedback comments and arguments [53].

This procedure is rarely used in medical education research (e.g. [11], [12]). More often, items are formulated in advance. The participating experts are asked to evaluate these items and suggest further ones. This can speed up the Delphi process. Additionally, the experts gain an understanding of what is expected of them and are more likely to generate comparable new content. This reduces both the workload for the respondents and the likelihood of them deviating from the topic. The development of the items included in the first survey round depends on the available resources [29]. For certain questions, statements can be taken from lists compiled by professional associations or committees. Often, relevant publications are available, enabling the steering group to compile the items based on the literature. If these requirements are not met, the steering group can develop the items directly. In this case, it is advisable to consult additional experts from specialist committees and bodies, either in person before the Delphi survey or via a preliminary survey.

#### 2.3.2. How is the number of rounds determined?

This should be decided at the start of the study, regardless of whether a consensus has been reached on all items. If achieving consensus is defined as a stopping criterion, the survey will continue until all items have been clearly assigned. Therefore, the effort involved cannot be calculated for either the steering group or the participants. 

The most important factors to consider when deciding how many rounds there will be are: how well the topic has already been researched, how heterogeneous the panel of experts is, how the consensus is defined, what result is to be achieved, and what resources are available. For example, Ellaway et al. [10] conducted a Delphi study to develop a curriculum for medical students on providing care to transgender and gender-diverse patients. The complexity of the topic required four rounds. However, good results can often be achieved in just two rounds (e.g. [54], [55], [56]).

#### 2.3.3. How is consensus measured?

In Delphi surveys, consensus means that a certain level of agreement is reached among the experts surveyed on a statement. However, it does not imply that all experts share the same opinion. In most publications, consensus is defined as being reached when 70-85% of experts agree with a statement [57]. Agreement is usually measured using descriptive statistical measures and depends on the scale level [58]. At a nominal scale level, the relative frequency of mentions can be determined. Typically, the degree of agreement is measured on an ordinal scale. Here, too, it is possible to determine the proportion of respondents who agree or disagree with or reject a statement once a certain characteristic threshold is reached. The median and interquartile range (IQR) are used more frequently because they allow majorities to be depicted [59]. For example, in a 5-point rating scale, an IQR <1 can be defined as consensus [58]. The mean and standard deviation should be avoided for ordinal scales. Consensus can also be determined using ranking questions. However, it should be noted that, despite requiring more cognitive effort from respondents, ranking questions generally offer no additional benefit over rating questions [60]. 

#### 2.3.4. How should the first questionnaire be structured?

The first Delphi round questionnaire should open with a preamble and collect demographic data. It should also contain a central section comprising both open-ended and closed-ended questions. The scale levels and options for the closed-ended questions must be chosen carefully [28], [59], [61] (see table 3 (Tab. 3)). 

A clear structure to the first questionnaire and an easily manageable amount of collected data are key to ensuring that follow-up rounds and feedback are not overloaded and can be appropriately designed [30]. Belton et al. recommend that the processing time for a Delphi round should not exceed 30 minutes, and that the first round should only include a few open-ended questions [62].

#### 2.3.5. How should communication with experts be handled?

It is important to treat the participating experts as partners and to explain all important steps. The process begins with clearly identifying the area of expertise. This means that interviewees should not be referred to as experts until the term has been defined [30]. The request for participation should be personalised. Explaining why their expertise is important increases their willingness to participate. To maintain motivation, social rewards are often more effective than financial ones [62]. For example, participants may be motivated if they recognise that their answers influence the outcome of the Delphi survey, or if they are provided with background information and interim results. Furthermore, using the appropriate language in the questionnaire and accompanying materials is crucial for success. In the case of heterogeneous expert panels, such as those in international studies or participatory Delphi surveys, it is particularly important to involve representatives from each subgroup of the expert panel in the pretest, to ensure the questionnaire is comprehensible to all participants [63].

### 2.4. Step 4: Evaluation strategies and planning the follow-up rounds

#### 2.4.1. Which strategies are used for the analysis?

The evaluation strategy depends on the type of data available. Quantitative data is typically analysed using descriptive statistics. For consensus measurement, each item is considered individually and placed into groups based on defined consensus criteria: *Consensus of agreement, dissent, or consensus of disagreement.*

Responses to open-ended questions are analysed according to their function [64] (see table 3 (Tab. 3)). Exploratory questions can be categorised and summarised using either quantitative content analysis [65] or thematic analysis [66]. Questions on extension can be analysed using quantitative content analysis [67]. Evaluating qualitatively recorded arguments in which interviewees justify their quantitative evaluations plays a special role. The five-stage, *Argument-based QUalitative Analysis* (AQUA) method was developed specifically for this purpose. This method enables qualitative data to be evaluated in a structured way and prepared for feedback alongside quantitative data [68].

#### 2.4.2. How is feedback transmitted? 

After each Delphi round, the participants should receive an objective feedback report on the aggregated group results. The group composition should be presented anonymously. Quantitative data can be visualised using position and scatter measures, e.g., bar or column charts. Individualised feedback, where each participant's evaluation is compared to the group values, is usually time-consuming. Its usefulness is often questioned, as it creates pressure to conform and increases cognitive load [69]. 

**Figure 1:**
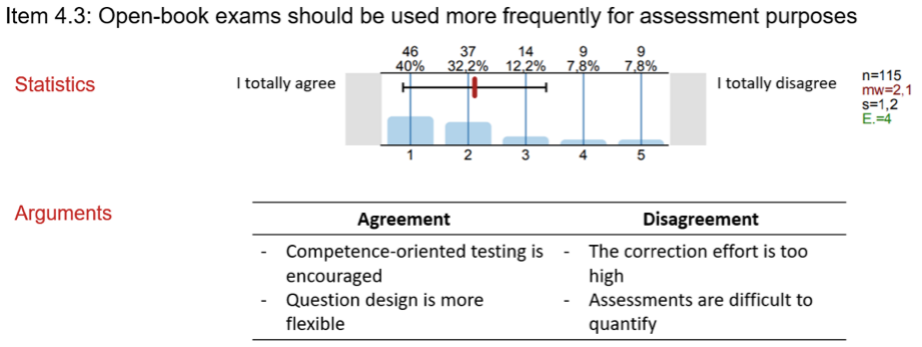
Example of feedback design in Delphi surveys

The qualitative results should be well structured and remain close to the original wording, enabling the experts to reflect on their contributions. The aim is to encourage participants to reconsider their views in light of the group responses and potentially adjust them. Figure 1 (Fig. 1) illustrates how quantitative data can be presented concisely alongside individual feedback and arguments. 

The feedback report can either be sent to respondents as a separate document alongside the invitation to the follow-up round or integrated into the follow-up questionnaire.

#### 2.4.3. How is the determined consensus dealt with? 

Items for which a consensus of agreement or disagreement has been reached are presented in the interim and final reports, but will not be re-evaluated in the follow-up round. Although a new vote could determine the reproducibility and stability of the ratings, the additional burden on respondents usually outweighs this advantage.

**Figure 2:**
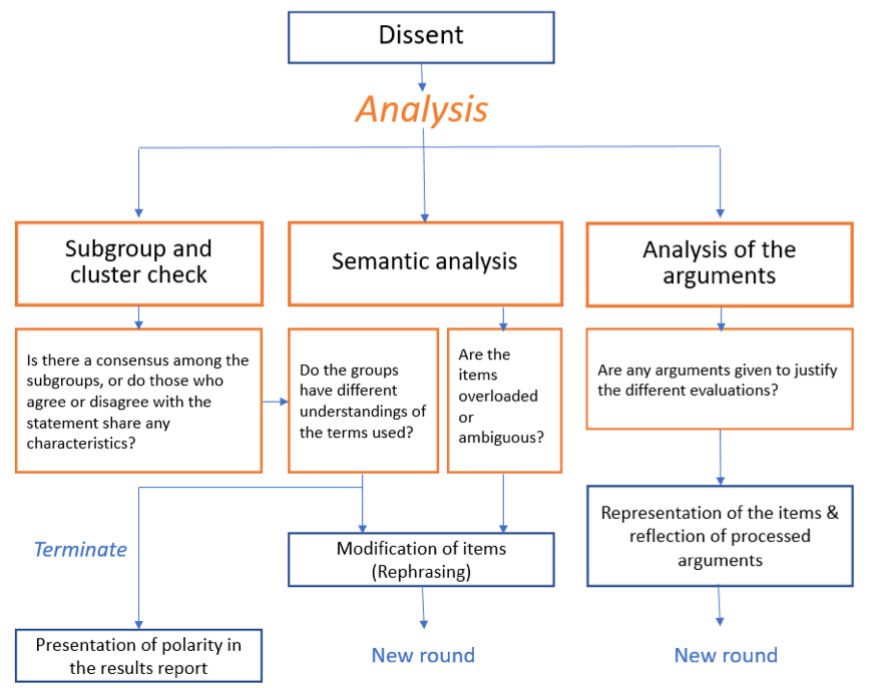
Analysis techniques for items that remain in disagreement in Delphi surveys

Items for which no consensus was reached regarding agreement or disagreement are analysed [70]. Depending on the outcome of this analysis, the items may be terminated, modified or resubmitted unchanged alongside the evaluation arguments [36] (see figure 2 (Fig. 2)). 

#### 2.4.4. How are the follow-up questionnaires structured?

Like the initial questionnaire, the follow-up questionnaires contain identical or modified items from the previous round. They also include items generated from exploratory questions. Furthermore, experts should have the opportunity to justify their judgements. Open-ended exploratory questions should not be included in the further course of the Delphi survey, or only to a limited extent.

### 2.5. Step 5: Presentation and dissemination of the results

#### 2.5.1. What about items that remain in dissent? 

Even after many rounds, it is possible that no consensus has been reached on individual items. This must be addressed and analysed. 

For example, it could be that the experts:

- generally have different priorities for values or goals,
- weight or consider partial aspects differently, or
- have conflicts of interest and therefore cannot deviate from their positions.

The cause may also lie in the methodological implementation. For example:

- the topic area may not have been narrowed down sufficiently in advance,
- arguments were not communicated or taken into account sufficiently in the feedback,
- the number of rounds was insufficient, or
- the items may not have been formulated precisely during the process.

Any remaining disagreements can be discussed further within the steering group, in a final meeting with the participating experts, or with the involvement of other experts or stakeholders. In any case, dissenting opinions should be included in the results report.

#### 2.5.2. How are the results published?

In order to increase the quality and transparency of reporting, a corresponding reporting guideline should be used. DelphiSTAR (Delphi studies in social and health sciences - Recommendations for an interdisciplinary standardized reporting) was developed specifically for publications in the health and social sciences [35]. Alternatively, one could use ACCORD (ACcurate COnsensus Reporting Document), developed for biomedicine, or CREDES (Guidance on Conducting and Reporting DElphi Studies), developed for the palliative care sector [31]. 

Participating experts contribute their experience and a considerable amount of time to the Delphi process. This should be acknowledged, and, if consent has been given, the experts should be named. Their qualifications can be presented in an attachment to illustrate their contributions to the Delphi process or to disclose conflicts of interest.

#### 2.5.3. How are the results disseminated and implemented?

Even before beginning the study, researchers should consider whether measures are necessary to disseminate and implement the results. Often, the results must be processed further before they can be put into practice. For instance, summaries can be created and distributed to target groups. A final workshop can also be helpful for this purpose (e.g. [71], [72]). Depending on the outcome, it may be useful to evaluate the results’ feasibility in practice and examine further application and implementation possibilities.

## 3. Discussion

This article provides a concise guide to conducting Delphi surveys in medical education research. It outlines key steps, explains standard procedures and alternatives, and highlights potential pitfalls. Readers are expected to have a basic understanding of the principles of empirical social research, including the construction of questionnaires, the operationalisation of theoretical concepts, and the analysis and interpretation of data. Researchers wishing to use Delphi variants must familiarise themselves with the relevant in-depth literature.

It is important to remember that methodological decisions often have far-reaching consequences. For instance, the composition of the expert panel [29], the choice of rating scale, and how consensus is determined [59] can all significantly impact the results. The statistical presentation of the results may mislead people into interpreting them as objective measurements and overestimating their significance. In medical education research, however, they are better understood as the result of a negotiation process in a given situation, whereby an agreement is reached on how something should or could be done. A major challenge is that many issues relating to Delphi surveys remain unresolved. For example, there is a lack of secondary analyses, experimental studies, or methodological tests that would allow for well-founded conclusions to be drawn about the influence of methodological and statistical procedures on judgement behaviour and, consequently, on consensus building [3], [58]. Nevertheless, this method is the preferred way to systematically bring together the specialised knowledge and real-life experience of experts from science and practice to increase certainty in planning and action. This guide can help to improve the quality of Delphi studies in medical education research. The steps and recommendations mentioned here can largely be transferred to other Delphi studies in health and educational sciences.

## Author’s ORCID

Angelika Homberg: [0000-0001-5585-1126]

## Competing interests

The author declares that she has no competing interests.

## Supplementary Material

Checklist for conducting Delphi studies in medical education research

## Figures and Tables

**Table 1:**
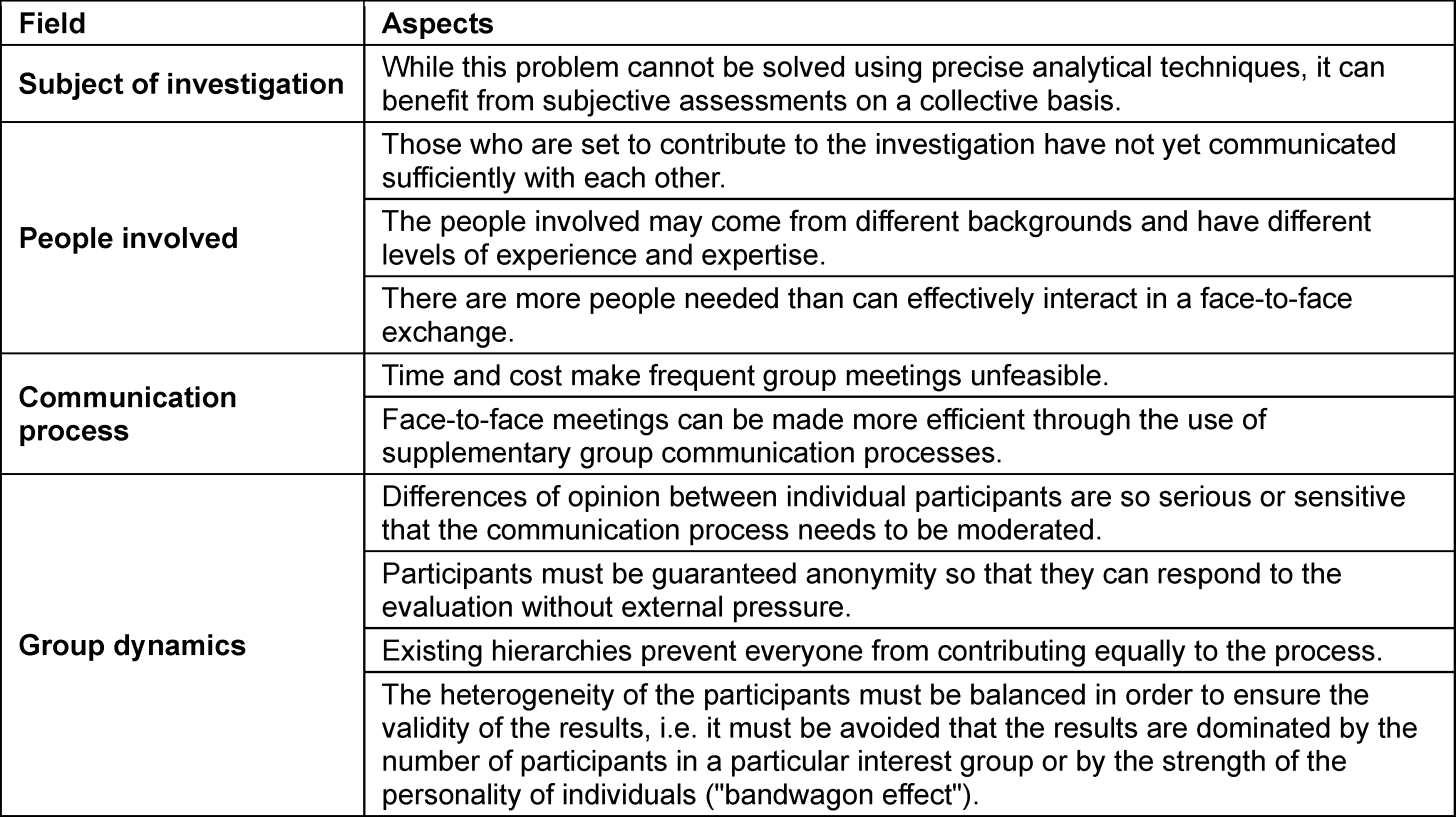
Aspects that lead to the application of the Delphi method (based on Linstone & Turoff 1975 [36])

**Table 2:**
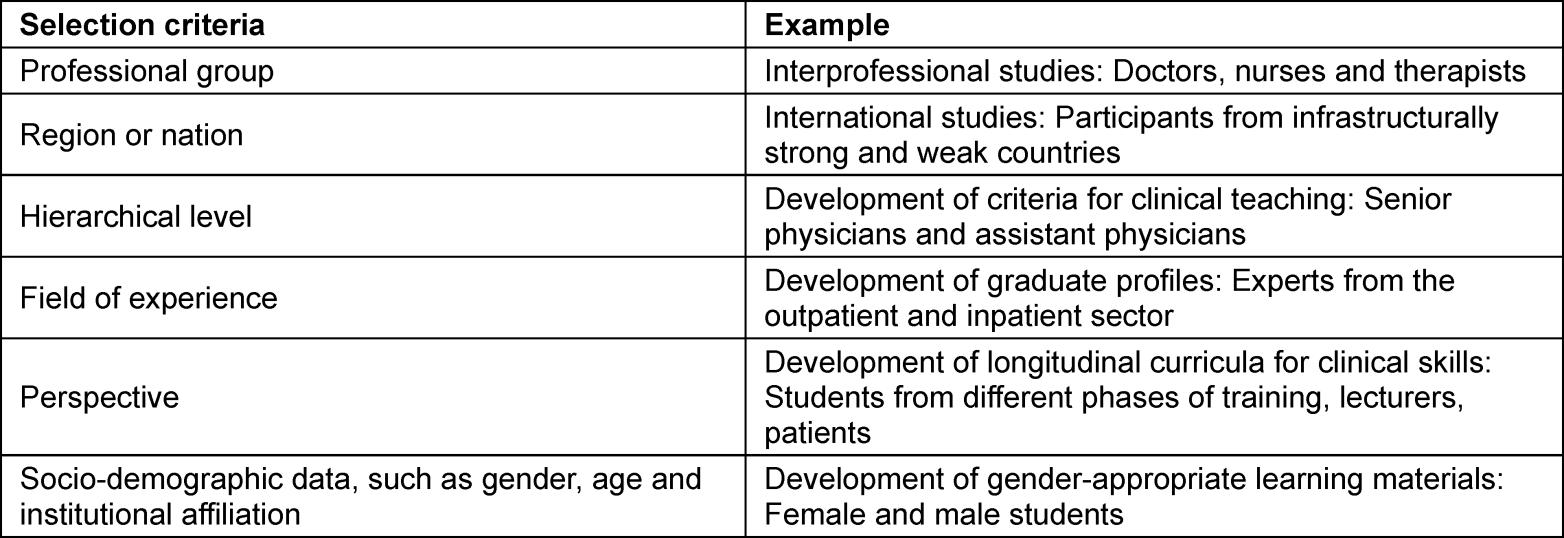
Balanced composition of the expert panel depending on the objective of the Delphi survey

**Table 3:**
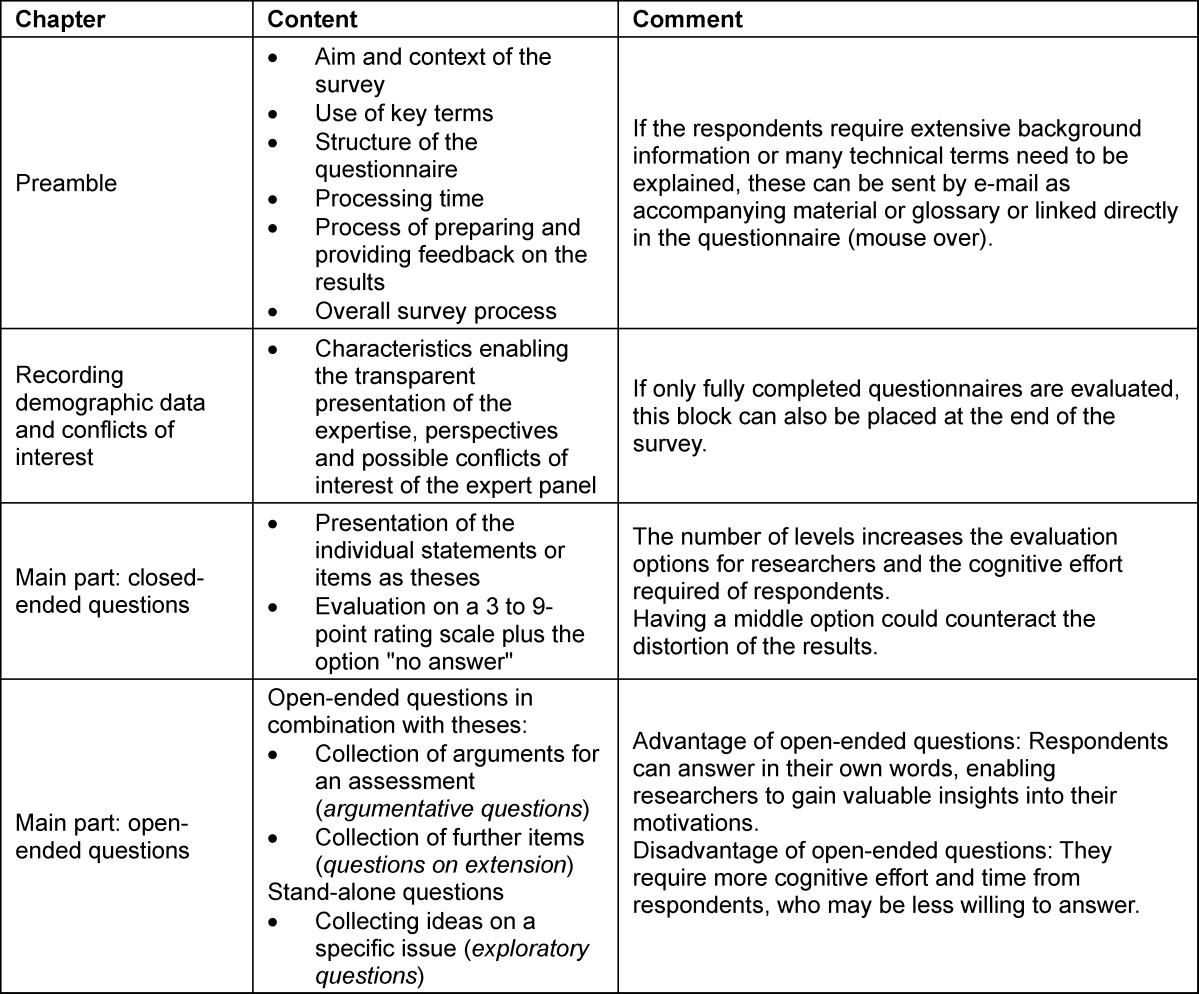
Structure of the questionnaire for the first Delphi round
