# Supplementary material for: Conducting Delphi surveys in medical education research
Source: GMS J Med Educ. 2026 Jan 15;43(1):Doc6. doi: 10.3205/zma001800 (PMC12875060; doi:10.3205/zma001800)
Supplement: Checklist for conducting Delphi studies in medical education research [file JME-43-6-s-001.pdf]

Attachment 1: Checklist for conducting Delphi studies in medical education research

Step	Item		Status
**1. Suitability and feasibility of the method**	1	The research problem and the aim of the study were clearly defined.	
	2	Various possible communication strategies for solving the problem were considered.	
	3	Other research methods would be less effective in achieving the objective.	
	4	There is sufficient time to conduct two or more rounds of the survey.	
	5	The researchers have the necessary human resources to complete the tasks between individual survey rounds in a timely manner.	
	6	Suitable survey software is available or can be purchased.	
	7	The potential costs have been clarified and the financing has been arranged.	
**2. Research question and persons involved**	8	A steering group has been assembled.	
	9	The steering group has the necessary methodological expertise to plan and manage the Delphi process.	
	10	The distribution of tasks within the steering group has been defined.	
	11	The planning steps and decisions of the steering group are being recorded.	
	12	The research objective has been reviewed and specified.	
	13	The steering group has agreed on the scope and context with regard to the validity of the research results.	
	14	It was determined<br>... whether the Delphi method was intended to achieve a consensus or another objective.	
	15	... what understanding the term "expert" is based on and how it will be used in the study.	
	16	... which criteria were used to compile the expert panel.	
	17	... how many people are to be interviewed per group.	
	18	... which criteria are used to recruit the individual experts.	
	19	The available resources of the steering group were taken into account during the planning process.	

Attachment 1 to Homberg A. *How to: Conducting Delphi surveys in medical education research.* GMS J Med Educ. 2026;43(1):Doc6. DOI: 10.3205/zma001800

Step	Item		Status
**3. Planning the survey process up to the first round**	20	It was determined<br>... how the items for the first round will be developed.	
	21	... how many rounds the Delphi should have.	
	22	... how long each Delphi round would last.	
	23	... how consensus is measured.	
	24	... how the questionnaire is structured.	
	25	... what information is included in the preamble and whether additional materials are provided.	
	26	... which demographic data and conflicts of interest are collected.	
	27	... which scales are used for standardized items.	
	28	... which aspects are recorded with open-ended questions.	
	29	... how high the proportion of open-ended questions is overall.	
	30	... when and how a pretest is carried out.	
	31	... how it is ensured that the questionnaire is understandable for the participants.	
	32	... how the experts are invited to participate.	
	33	... what means can be used to encourage and maintain the motivation of the participants.	
**4. Evaluation on strategies and planning the follow-up rounds**	34	It was determined<br>… how the standardized items will be evaluated and presented graphically.	
	35	... which method will be used to evaluate and summarise the qualitative data.	
	36	... which elements the feedback report should contain.	
	37	... how the feedback report will be sent to the experts.	
	38	... how the arguments that remained in disagreement are taken up in the follow-up rounds.	
**5. Presentation and dissemination of the results**	39	It was determined<br>… how to deal with items remaining in dissent at the end of the Delphi survey.	
	40	... how the results are to be published.	
	41	... whether it is necessary to obtain an ethics vote.	
	42	... which reporting guideline will be used.	
	43	... whether and in what form the participants are named in the publication.	
	44	... whether a final workshop or other measures will be held to discuss the judgments that remain in dissent.	
	45	... whether further measures are necessary to process, disseminate and implement the results.	
